# Electrophysiological predictors of propafenone efficacy in prevention of atrioventricular nodal re-entrant and atrioventricular re-entrant tachycardia

**DOI:** 10.3325/cmj.2012.53.605

**Published:** 2012-12

**Authors:** Hrvoje Pintarić, Ivan Zeljković, Zdravko Babić, Mislav Vrsalović, Nikola Pavlović, Hrvojka Bošnjak, Dubravko Petrač

**Affiliations:** 1 Department of Internal Medicine, Sestre milosrdnice University Hospital Center, Zagreb, Croatia; 2 School of Dental Medicine, University of Zagreb, Zagreb, Croatia; 3 University of Zagreb School of Medicine, Zagreb, Croatia

## Abstract

**Aim:**

To assess the efficacy of propafenone in prevention of atrioventricular nodal reentrant tachycardia (AVNRT) and orthodromic atrioventricular tachycardia (AVRT) based on the clinical results of arrhythmia recurrence and find the electrophysiological predictor of propafenone effectiveness.

**Methods:**

This retrospective study included 44 participants in a 12-month period, who were divided in two groups: group A – in which propafenone caused complete ventriculo-atrial block and group B – in which propafenone did not cause complete ventriculo-atrial block.

**Results:**

Group A had significantly lower incidence of tachycardia than group B (95% vs 70.8%, *P* = 0.038), and complete ventriculo-atrial block predicted the efficacy of propafenone oral therapy in the prevention of tachycardia (sensitivity 87.5%, specificity 52.8%, positive predictive value 95%, negative predictive value 29.2%). Patients with AVNRT in group B who did not experience the recurrences of tachycardia had significantly shorter echo zone before intravenous administration of propafenone than the patients who experienced episodes of sustained tachycardia (median 40 ms [range 15-60 ms] vs 79 ms [range 50-180 ms], *P* = 0.008).

**Conclusion:**

In patients with non-inducible tachycardia, complete ventriculo-atrial block can be used as an electrophysiological predictor of the efficacy of propafenone oral therapy in the prevention of tachycardia. In patients with non-inducible AVNRT, but without complete ventriculo-atrial block, propafenone was more effective in patients with shorter echo zone of tachycardia.

Nowadays a leading method in treatment of supraventricular re-entrant tachycardias is radio-frequent (RF) catheter ablation (1-4). However, in some patients the method of choice in the prevention of tachycardia recurrence is treatment with antiarrhythmics (5). The medications used can be selected on the basis of the previous clinical experience or the results of the electrophysiological study (EPS) (6). A few groups of antiarrhythmics can be used in prevention of supraventricular re-entrant tachycardias, atrioventricular nodal re-entrant tachycardia (AVNRT) and atrioventricular re-entrant tachycardia (AVRT), such as: class IV (verapamil), Ic (propafenone and flecainide), and II (beta-blockers) (7-9). It is extremely important to establish the characteristics of patients’ clinical tachycardia, as well as the echo zone of the tachycardia (10,11). The medicines effective in patients with long-lasting echo zone are class Ic antiarrhythmics (12,13), and in patients with shorter echo zone potassium channel blockers (sotalol, amiodarone), due to their strong effect on the refractory period of the accessory pathway (9,14). Propafenone is a class Ic antiarrhythmic agent, with mild beta-blocking activity, affecting both pathways of AVNRT and orthodromic AVRT, thus causing retrograde or antegrade block (9). It has been extensively used in the treatment of a number of supraventricular tachyarrhythmias (7,15). In our previous study, we examined the safety and efficacy of intravenous propafenone in termination of both AVNRT and AVRT, as well as the electrophysiological effects of propafenone on cardiac conductivity (16).

The aim of this study was to examine the effectiveness of propafenone in the prevention of tachycardia (AVNRT and AVRT) recurrence with regard to several electrophysiological parameters. This has been done in previous similar studies, but they included fewer patients and electrophysiological findings were not evaluated as predictors of antiarrhythmic agent effectiveness (17,18).

## Patients and methods

### Design and settings

This retrospective, single-center study included 44 patients who had previously undergone electrophysiological study (EPS) followed by intravenous administration of propafenone at the Department of Cardiology, Sestre Milosrdnice University Hospital Center, Zagreb, Croatia between January 2004 and December 2005 and continued to receive propafenone oral therapy during a 12-month period after the EPS. These 44 patients were chosen from all the patients (70 patients) who underwent the electrophysiological procedure at the University Hospital Centre Sestre Milosrdnice, Department of Internal Medicine between January 2004 and December 2005 and among whom tachycardia could not be induced after iv. propafenone had been administered.

### Electrophysiology study protocol and intervention

The study included a total of 44 patients with symptoms of two most common suparaventricular tachycardias, typical AVNRT and orthodromic AVRT, documented by 12-lead ECG, with minimally four episodes per year during the last three years. After taking their complete medical history, each patient underwent a thorough clinical examination, routine laboratory testing, chest x-ray, standard ECG recording, and echocardiogram.

Since they were diagnosed with tachycardia, all patients were treated with different antiarrhythmic drugs ranging from one to five antiarrhythmics (group A: median 4, range 1-5; group B: median 4, range 1-5, *P* = 0.256). The most frequently used antiarrhythmic was atenolol (in 47 patients, 67.1%). Thirty-four patients (48.6%) were treated with verapamil, 29 (41.1%) with sotalol, and 28 (40.0%) with amiodarone. Eight patients were previously treated with propafenone (11.4%), 4 with digoxin (5.7%), and one with disopyramide (1.5%). All antiarrhythmic drugs were discontinued at least 30 days before the EPS (19,20).

All patients underwent EPS according to standard protocol (19-21), using four 6-French, quadripolar electrodes positioned under fluoroscopy in the right atrium, right ventricular apex, coronary sinus, and bundle of His. Conduction intervals and refractory periods for EPS were defined in the conventional manner (19). Before starting the EPS, a written informed consent was obtained from each patient.

Tachycardia was induced by standard stimulation protocols using a Mingograph 62 Siemens stimulator (Elema AB, Stockholm, Sweden). Programmed atrial stimulation was the most frequent way of inducing tachycardia, whereas fast atrial and ventricular stimulation was the most frequent way of terminating tachycardia. Programmed ventricular stimulation induced tachycardia in 12 patients with orthodromic AVRT (36.4%) and 4 patients with AVNRT (10.8%), with the difference being significant (*P* = 0.011, *t* test proportion for independent samples). There was no significant difference in other types of tachycardia induction, which was induced easily by all applied methods (*P* > 0.05, *t* test proportion for independent samples).

Two techniques are used to evaluate retrograde ventriculo-atrial (VA) conduction in the evaluation of SVTs: burst pacing in the right ventricle and premature ventricular stimulation. Retrograde properties were evaluated by noting the longest ventricular paced cycle length that produced VA block and by measuring the ERP period of VA conduction (20).

The antegrade echo zone is defined as the zone of A1A2, at which A2 provokes atrial echoes with or without tachycardia. The average atrial echo zone is an interval from spontaneous atrial activity to atrial premature depolarization producing tachycardia (20,21).

The successful induction of tachycardia and determination of its mechanism were followed by intravenous administration of propafenone (2 mg/kg over 10 minutes) (19,20). The same electrophysiological protocol was repeated to assess tachycardia inducibility. During the procedure, vital parameters including blood pressure, ECG, and oxygen saturation were monitored. After the completion of EPS, standard ECG was recorded and compared to the one obtained prior to propafenone administration.

### Study protocol

The exclusion criteria for EPS and intravenous administration of propafenone were the following: bradycardia, AV block, sinus node dysfunction, hypotension, coronary artery disease, cerebrovascular disease, left ventricular hypertrophy, reduced left ventricular systolic function, renal or liver failure, hypokalemia, and pregnancy.

After the completion of EPS, each patient was prescribed individualized per os antiarrhythmic therapy aimed to prevent tachycardia. Forty-four participants were included into a retrospective study after a 12-month period after EPS and were divided in two groups: group A – in which propafenone caused complete VA block and group B – in which propafenone did not cause complete VA block.

Propafenone was considered to be a drug of choice as continuous oral therapy in patients in whom tachycardia was non-inducible after its intravenous administration during the EPS. In patients in whom iv. propafenone during EPS did not prevent tachycardia (22/66 patients, 33%) per os therapy with atenolol, sotalol, or amiodarone was prescribed.

Patients were divided into two groups according VA block because it was shown that completing VA block could represent a significant electrophysiological predictor of propafenone efficiency in tachycardia prevention (16,22,23).

During the period of 12 months, patients had regular controls every 3 months, which included medical history taking, physical examination, ECG recording, and 24-hour continuous ECG recording. Our focus was on the recurrence of clinical tachycardia, that is, on the efficacy of propafenone in its prevention, as well as possible side-effects of propafenone. If the patient was asymptomatic during this period, propafenone was considered to be effective. Recurrence of clinical tachycardia required discontinuation of propafenone. Moreover, several electrophysiological parameters were identified as predictors of a good clinical outcome (absence of tachycardia).

### Statistical analysis

Quantitative data are presented as median values and ranges and qualitative data by contingency tables as absolute values and percentages. The differences in quantitative variables between the groups were tested by Mann-Whitney U-test (non-parametric test for independent samples). The relation between qualitative variables and patients' groups was tested by χ^2^ test with Yates corrections for Tables 2 × 2. The differences in frequencies of certain variables between the groups were tested by *t* test proportion for small independent samples. The differences in quantitative variables before and after drug treatment were tested by Wilcoxon test for pair samples (non-parametric test for dependent samples). The effect of certain electrophysiological parameters on the prevention of tachycardia by propafenone treatment was evaluated on the basis of sensibility and reliability test. The effect of variables on relative risk of the prevention of tachycardia by propafenone treatment was evaluated by logistic regression and presented as odds ratio (OR) with 95% confidence intervals (CI). Statistical analysis was carried out using the Statistica 6.0 software (24). A value of *P* < 0.05 was considered statistically significant.

## Results

The study included 44 participants who continued to receive propafenone oral therapy (450-900 mg) after they had undergone EPS. Patients in group A received on average 669 ± 79 mg of propafenone in comparison with patients in group B who received 678 ± 168 mg (*P* = 0.569). After a period of one year, 36 patients (82%) continued to receive propafenone therapy, which was prescribed if tachycardia was non-inducible after its intravenous administration during the EPS. Nineteen patients in group A (95%) were asymptomatic and continued to receive antiarrhythmic therapy (propafenone), whereas 1 patient had tachycardia and therapy was terminated. In group B, 7 patients (29%) had palpitations leading to the termination of recommended propafenone therapy, while 17 patients (71%) were asymptomatic (Table 1). Group A had significantly lower incidence of tachycardia than group B (*P* = 0.038; *t* test proportion). Complete VA block predicted the efficacy of propafenone in the prevention of tachycardia (sensitivity 87.5%, specificity 52.8%, positive predictive value 95%, negative predictive value 29.2%) (Table 2).

**Table 1:** Demographic and clinical parameters in the group A (with complete ventriculo-atrial block) and group B (without complete ventriculo-atrial block)

Demographic and clinical data	Group A	Group B	*P*
	n = 20	n = 24	
**Age in years** (median, range)	44 (18-70)	47 (20-67)	0.301*
Symptom duration in months **(median, range)**	146 (44-450)	99 (38-480)	0.126*
**Sex,** n (%)**:**			
male	7 (35)	10 (42)	0.197^†^
female	13 (65)	14 (58)	
Heart disease (any, except those mentioned in exclusion criteria), n (%)	10 (50)	11 (46)	0.425^†^
Therapy (antiarrhytmics)	4(1-5)	4(1-5)	0.256*
Symptoms, n (%)			
presyncope	8 (40)	6 (25)	0.191^‡^
syncope	5 (25)	5 (21)	0.098^‡^
chest pain	0	1 (4)	0.234^‡^
palpitations	18 (90)	23 (96)	0.321^‡^

*Mann-Whitney U test.

†χ^2^ test.

‡*t* test.

**Table 2:** Sensitivity and specificity of complete ventriculo-atrial (VA) block as a predictor of efficacy of propafenone in prevention of tachycardia*

	Propafenone effective	Propafenone ineffective	Total
Tachycardia is non-inducible, complete VA block	19	1	20
Tachycardia is non-inducible, without complete VA block	17	7	24
**Total**	**36**	**8**	**44**

*Sensitivity = 87.5%; specificity = 52.8%; positive predictive value = 95%; negative predictive value = 29.2%.

Despite recommendations, 6 out of 22 patients in whom iv. propafenone during EPS did not prevent tachycardia (27%, 2 with AVNRT and 4 with AVRT) continued with the propafenone therapy. One out of 6 patients during the monitoring period of per os propafenone therapy (2 × 300 mg/per day) did not show the symptoms of tachycardia. Propafenone therapy was interrupted for the remaining 5 patients considering the recurrence of tachycardia.

After the patients with AVNRT in the group B (16 patients) were studied in isolation, we noticed that 11 patients (69%) did not have recurrent tachycardia. Compared with patients with AVNRT who had palpitations (5/16 patients, 31%), they had shorter echo zone during EPS before the intravenous propafenone administration, 40 ms (15-60 ms) vs 79 ms (50-180 ms) (*P* = 0.008) .

Side-effects of propafenone therapy occurred in 9 out of 44 patients (21%) and included the following: mild gastrointestinal upset, blurred vision, and constipation. However, there was no need to discontinue propafenone in any patient because of the side-effects. No patient showed proarrhythmic effect.

## Discussion

The retrospective, single-center study studied a sample of patients with the two most common supraventricular tachycardias: AVNRT and AVRT, which have considerable incidence in the general population, especially women (25,26).

In the study, propafenone was effective in the prevention if clinical tachycardia was absent during a one-year period after the EPS and the oral propafenone therapy was introduced. Among the patients with non-inducible tachycardia after intravenous administration of propafenone during the EPS, propafenone was more effective in the prevention of tachycardia in patients with achieved complete VA block. Therefore, the complete VA block can be used as an electrophysiological predictor of propafenone efficacy in the prevention of tachycardia recurrence.

Furlanello et al performed transesophageal EPS in 58 patients, with administration of class Ic antiarrhythmics (propafenone and flecainide) (27). Their study showed that these medications were extremely effective in the prevention of supraventricular tachycardias, if arrhythmia was non-inducible during the EPS after the drug application, with effectiveness of prevention of 65.5%, which is less than in our study (81.8%). Breithardt et al reported that propafenone was a very potent agent in the prevention of supraventricular tachycardias in patients with Wolf-Parkinson-White syndrome, independently of the results of EPS (28).

It is noteworthy that in adults with supraventricular re-entrant tachycardias, RF ablation during EPS has become the first-line treatment approach with a high acute success and low complication rate (3,4,29,30). However, Vassilikos et al showed that non-cardiologists (general practitioners and internists) who are involved in the acute and long-term management of SVT rely more on antiarrhythmic drugs and tend to underestimate the role of ablation therapy for the long-term management of SVT (31). RF ablation therapy is increasingly used in pediatric patients with SVTs, however, antiarrhythmic therapy is still the first choice (usually guided on the results of transesophageal atrial pacing technique), in which propafenone is one of the first choices and has good efficacy in termination of tachycardias (32,33). All this points to the importance of antiarrhythmics for the treatment of supraventricular tachycardias, and therefore to the importance of propafenone, which has proven to be efficient in our study as well as several previous studies, although they included a small number of patients (23,27,28). According to the literature overview, indication for continuous antiarrhythmic therapy is non-inducibility of tachycardia after antiarrhythmic agent administration (15,17,18).

Regarding the effectiveness of other groups of antiarrhythmics, Komatsu et al tested the effectiveness of four antiarrhythmics on the induction and termination of supraventricular re-entrant tachycardias (22). Verapamil was effective in about 65% patients, procainamide in somewhat more (around 75%), whereas propranolol and disopyramide were not particularly effective (under 40% overall). Our study showed better effectiveness for propafenone.

In addition, we tried to find the electrophysiological parameter that predicted propafenone effectiveness in patients in whom the complete VA block during EPS was not achieved. According to our results, in patients with AVNRT and without achieved complete VA block, propafenone was more effective in the prevention of tachycardia in patients with shorter echo zone of tachycardia prior to intravenous propafenone administration during EPS.

There are certain limitations of the study: it is a retrospective single-center study performed on a small group of patients. Moreover, the data are largely descriptive and due to the small number of patients, statistical analysis could not be performed between certain groups.

In conclusion, oral therapy with propafenone is more effective in the prevention of tachycardia recurrence in patients with achieved complete VA block during EPS. Complete VA block, achieved after intravenous administration of propafenone during the EPS, can be used as an electrophysiological predictor of efficacy of propafenone in the prevention of tachycardia recurrence.
